# Supplementary material for: Evaluation of a novel approach to community health care delivery in Ifanadiana District, Madagascar
Source: PLOS Glob Public Health. 2024 Mar 12;4(3):e0002888. doi: 10.1371/journal.pgph.0002888 (PMC10931426; doi:10.1371/journal.pgph.0002888)
Supplement: S1 Text — (DOCX) [file pgph.0002888.s003.docx]

Evaluation of a novel approach to community health care delivery in Ifanadiana District, Madagascar

**S1.** Interrupted time series analysis

To assess the impact of the intervention on monthly consultations by CHWs with children under-five, we conducted a supplementary analysis using an interrupted time series analysis. We modeled the number of under-five consultations per month in the intervention commune. The model was specified as:

$$Y_{t} = \beta_{0}+ \beta_{1}time + \beta_{2}intervention +\beta_{3} postinterventiontime$$

Where time is month since the start of the observation period (1-36), intervention indicates when the intervention started, and postinterventiontime is the count of months since the start of the intervention. As in the main body of the manuscript, we determine that the intervention positively impacted the number of monthly consultations.

|  | **Coefficient** | **Standard error** | **p-value** |
| --- | --- | --- | --- |
| Intercept | 101.8824 | 66.20273 | 0.1336 |
| Time in months | 4.5738 | 6.11609 | 0.4600 |
| Intervention | 333.6784 | 89.95713 | 0.0008 |
| Post-intervention time in months | -2.6440 | 8.64946 | 0.7618 |
