# Supplementary material for: Evaluation of a novel approach to community health care delivery in Ifanadiana District, Madagascar
Source: PLOS Glob Public Health. 2024 Mar 12;4(3):e0002888. doi: 10.1371/journal.pgph.0002888 (PMC10931426; doi:10.1371/journal.pgph.0002888)
Supplement: Attachment — Submitted filename: ECH evaluation_response to reviewers.docx [file pgph.0002888.s004.docx]

November 1, 2023

Dear Editor,

Thank you for providing thoughtful reviews of our manuscript. Besides a number of editorial considerations, the main thrust of the comments were relatively minor, asking to further clarify details of the community health program in this study, and how it differed from comparison programs in both services and costs. In addition, there was a suggestion on the analysis of data itself. We have revised the manuscript to address this feedback, including providing a supplementary interrupted times series analysis, and hope that you find that these revisions have strengthened the manuscript. All of the findings on the effectiveness of the enhanced community health program in Ranomafana, Madagascar, remain the same, statistically significant improvements in nearly every CHW performance indicator. Itemized responses to the Editor’s comments are below:

1. Please amend your detailed online Financial Disclosure statement. This is published with the article. It must therefore be completed in full sentences and contain the exact wording you wish to be published.

We have amended the funding statement to respond to the editor’s request.

2. Please declare all competing interests beginning with the statement "I have read the journal's policy and the authors of this manuscript have the following competing interests:"

Thanks, we have clarified the statement on competing interests.

3. Please provide a complete Data Availability Statement in the submission form, ensuring you include all necessary access information or a reason for why you are unable to make your data freely accessible. If your research concerns only data provided within your submission, please write "All data are in the manuscript and/or supporting information files" as your Data Availability Statement.

We have revised the Data Availability Statement in accordance with ethics committee data sharing guidelines. As it now states, we will provide the data as we are able in accordance with those guidelines which involve Madagascar Ministry of Public Health Data. This update includes a response to the concern about copyright issues with the map.

4. Some material included in your submission may be copyrighted. According to PLOS’s copyright policy, authors who use figures or other material (e.g., graphics, clipart, maps) from another author or copyright holder must demonstrate or obtain permission to publish this material under the Creative Commons Attribution 4.0 International (CC BY 4.0) License used by PLOS journals. Please closely review the details of PLOS’s copyright requirements here: PLOS Licenses and Copyright. If you need to request permissions from a copyright holder, you may use PLOS's Copyright Content Permission form.

We have added information on the data availability of the map as part of the updated Data Availability Statement. As it says now, the map comes from OpenStreetMap and is authorized to be shared with acknowledgement.

5. Please review your reference list to ensure that it is complete and correct. If you have cited papers that have been retracted, please include the rationale for doing so in the manuscript text, or remove these references and replace them with relevant current references.

We have reviewed the reference list to ensure that information is updated and complete.

6. In Table 1, is there a way to highlight the key differences between the columns for each row?

Yes, thanks for the suggestion. We revised Table 1 to make it easier to distinguish differences in the programs, including bolding key features of the expanded community health program, and we adjusted the title to highlight this difference.

7. The costing evidence is valuable enough that I would propose you move it from the discussion to the results section (and potentially add some description of the approach to gathering this data to the methods section).

Thank you for this feedback. We have moved the costing findings to the results section, and accordingly amended the methods section to describe where data on costs were derived.

In response to the reviewer, we have adapted the manuscript as follows:

*1. One limitation of the study is that the quasi-experimental design cannot establish causality, and other factors besides the ECH model may have contributed to the observed improvements in utilization and quality of care. I think the study can be strengthened by additional examination such as performing an interrupted time series analysis with control to rule out alternative explanations for observed changes in the dependent variable over time instead of only conducting t-test and chi-squared test.

Other suggestions or questions:

1. The findings can be enhanced by quantifying the effects of intervention through a difference-in-difference analysis.

2. It would be helpful to conduct an equivalent regression analysis to produce uncertainty estimates on the results. E.g., line 34.

3. Figure 1 shows that the health utilization of both intervention and comparison group has increased after the intervention was implemented, is there any explanation for that? Using an interrupted time series design with a control group could increase the validity of the study results.*

*4. It would be helpful to discuss other possible factors that might have contributed to the performance of the intervention group to increase.*

We have included a difference-in-differences analysis in the main body of the manuscript. The results section now includes a statement which reads, "Mean monthly consultations for children under-five increased significantly more for the ECH intervention area than for the comparison area (DID estimate 149.85 [95% confidence interval 49.3, 250.4], p-value <0.01)."

In response to these comments, we have provided additional information on possible factors in the discussion section. It now says, “Community health workers included in the ECH intervention demonstrated improvements in utilization and quality of care. It is not possible to isolate effects of any single element of the program on outcomes. It is possible that these changes were influenced by increasing familiarity with protocols over time and not program changes. Additionally, as supervision included support on record-keeping and data submission, it is possible that changes over time are a reflection of better reporting by CHWs. Supervision included support for reporting in both ECH and comparison communes.”

5. Is it possible that the recruitment of more skillful CHWs from the comparison commune to the intervention commune, due to higher compensation, have caused the differences in outcomes between the two groups?

This is a valid question. Thanks for mentioning this. To address this, we have added a table to the Supplementary Information which compares quality measures between experienced CHWs and those newly hired in the ECH intervention area. There are minimal differences between the two groups, which is now referenced in the discussion section of the manuscript.

6. Discussing and justifying why the control groups are comparable to the intervention group would be helpful.

Thanks for suggesting this. We have added a table which compares the intervention and comparison communes in a table included as part of the Supplementary Information. These data include measures of overall health system functioning, disease burden, and health system utilization.

7. Table 1 presents various health interventions evaluated in the program. To inform policy recommendations, it would be beneficial to discuss the individual contributions of each intervention. For instance, which intervention might have had a greater impact compared to others?

As mentioned above, given our study design, we are unable to identify the impact of individual program components on the outcomes of interest. Anecdotal responses from CHW feedback, suggests that training and consistent supervision play an important role in CHW performance. Moreover, under the new national Madagascar community health protocol, the financial incentive for CHWs will be reduced and community-based supervision frequency will change. This change will provide an opportunity to review the impact of these changes on utilization and quality. We look forward to pursuing this question further. We acknowledge the limitation of our current study in the discussion section by stating, “It is impossible to isolate if any single element of the program drove the improvements in outcomes.”

Thank you again for your careful consideration of our manuscript.
